# **Potential Mechanisms Underlying the Therapeutic Roles of *Gancao fuzi*** Decoction in Cold-dampness Obstruction Syndrome-type Knee Osteoarthritis

**DOI:** 10.2174/1573409919666230605115940

**Published:** 2023-10-09

**Authors:** Jinlong Zhao, Guihong Liang, Hetao Huang, Weiyi Yang, Jianke Pan, Minghui Luo, Lingfeng Zeng, Jun Liu

**Affiliations:** 1 The Second Clinical College of Guangzhou University of Chinese Medicine, Guangzhou, 510405, China;; 2 The Second Affiliated Hospital of Guangzhou University of Chinese Medicine Guangdong Provincial Hospital of Chinese Medicine, Guangzhou, 510120, China;; 3 The Research Team on Bone and Joint Degeneration and Injury of Guangdong Provincial Academy of Chinese Medical Sciences, Guangzhou, 510120, China;; 4 The Fifth Clinical College of Guangzhou University of Chinese Medicine, Guangzhou, 510405, China;; 5 Guangdong Second Chinese Medicine Hospital Guangdong Province Engineering Technology Research Institute of Traditional Chinese Medicine, Guangzhou, 510095, China

**Keywords:** Network pharmacology, *Gancao fuzi* decoction, cold-dampness obstruction syndrome, knee osteoarthritis, action mechanism, molecular mechanism

## Abstract

**Background:**

The key active components and potential molecular mechanism of *Gancao fuzi* decoction (GFD) in the treatment of cold-dampness obstruction-type knee osteoarthritis (KOA) remain unclear.

**Objectives:**

To explore the mechanism of GFD in the treatment of cold-dampness obstruction syndrome-type KOA by network pharmacology.

**Methods:**

The potential active components and targets of the four herbs in GFD (Fuzi, Guizhi, Baizhu, and Gancao) were screened using the Traditional Chinese Medicine Systems Pharmacology (TCMSP) database. The targets of KOA were obtained with the Comparative Toxicogenomics Database (CTD), the GeneCards database, and the DisGeNET database, and the common targets of the drugs and disease were ultimately obtained. Cytoscape (v.3.7.1) was used to draw the active component-target network, and the Search Tool for the Retrieval of Interacting Genes/Proteins (STRING) (v.11.0) database was used to construct the protein interaction network. The Database for Annotation, Visualization, and Integrated Discovery (DAVID) was used for the Gene Ontology (GO) function and Kyoto Encyclopedia of Genes and Genomes (KEGG) pathway enrichment analyses of the intersecting targets.

**Results:**

A total of 102 potential active components and 208 targets of GFD in the treatment of cold-dampness obstruction syndrome-type KOA were screened. GFD treatment was found to be closely related to many inflammatory signalling pathways in the treatment of KOA.

**Conclusion:**

The effect of GFD on cold-dampness obstruction syndrome-type KOA is mediated by multicomponent, multitarget, and multichannel mechanisms, which provides the basis for further experimental study of its pharmacodynamic material basis and mechanism.

## 1 INTRODUCTION

Knee osteoarthritis (KOA) is a common chronic degenerative disease and is mainly caused by mechanical and biological factors that lead to normal articular chondrocyte apoptosis and extracellular matrix degradation [1, 2]. The main clinical symptoms of KOA are pain, stiffness, and joint swelling, which is one of the main causes of joint deformity. The disease is common in middle-aged and elderly people (over 50 years of age), has a prevalence rate among people over 60 years of age as high as 50%, and mainly occurs in female patients [3, 4]. In the United States, more than 10 million people suffer from KOA every year, and the annual cost of the treatment of the disease is as high as $420 million [5, 6]. To delay the end-stage development of KOA, the commonly used conservative treatment schemes mainly include oral nonsteroidal anti-inflammatory drugs, intra-articular injections of hyaluronic acid or platelet-rich plasma, and physical therapy, but the curative effect remains uncertain [7, 8]. Therefore, exploring new drug therapies for the prevention and treatment of KOA is particularly important.

*Gancao fuzi* decoction (GFD) originates from a treatise on *Shanghan Lun* written by *Zhang Zhongjing* and is composed of four herbs: Fuzi, Guizhi, Baizhu, and Gancao. GFD has the effects of dispelling, and removing dampness, warming the meridian, dispersing cold, warming yang, and tonifying the middle. This prescription is famous for the treatment of cold-dampness obstruction-type KOA. Cold-dampness obstruction syndrome is a type of KOA in basic traditional Chinese medicine (TCM). Dampness, an external evil, plays a key role in the pathological development of bone and joint diseases. In the clinic, all patients with *wind cold-dampness Bi* can flexibly use GFD [9]. However, due to the complex active components and multiple targets of GFD, the key active components and potential molecular mechanism of GFD in the treatment of cold-dampness obstruction-type KOA remain unclear.

The “one drug, one gene, one disease” model, often followed in new drug research and development, maybe one of the main reasons for the failure of many new drugs in clinical trials. A variety of clinical diseases, such as KOA, are multigene and multifactor diseases [10, 11], and achieving the expected effect using only a single-target therapy is difficult. Network pharmacology emphasizes the multitarget and multichannel regulation of signalling pathways, which can play an important role in the research and development of new drugs, particularly in innovative drug research and the development of TCM. Network pharmacology explores the correlation between drugs and diseases from a holistic perspective, consistent with the principles of TCM. In recent years, the research methods and ideas of network pharmacology have been widely used in TCM prescription research. The application of network pharmacology can allow a more systematic and accurate analysis of the broad component-target correspondence [12] and provide new approaches and strategies for the research and development of TCM. In this study, network pharmacology [13, 14] was used to explore the possible composition, signalling pathway, and target protein of GFD in the treatment of cold-dampness obstruction-type KOA to provide a theoretical basis for new drug development and clinical application.

## 2 MATERIALS AND METHODS

### 2.1 Screening of Active Components and Targets of GFD

The active ingredients of GFD were extracted from the Traditional Chinese Medicine Systems Pharmacology Database and Analysis Platform (TCMSP) [15]. The compounds were screened according to their oral bioavailability (OB) and drug-like (DL) properties. The screening conditions were OB ≥ 30% and DL ≥ 0.18, and the selected compounds were the core active components. The TCMSP was used to obtain the drug targets corresponding to the active ingredients, and the UniProt database [16] was used for standardized sorting, screening human targets, and correcting the target names.

### 2.2 Acquisition of Disease Targets in KOA

In the Comparative Toxicogenomics Database (CTD) [17], GeneCards database [18], and DisGeNET database [19], the disease targets related to KOA were collected using “osteoarthritis, knee” as the keywords, and the duplicate items obtained from the three databases were then removed to obtain the final disease targets.

### 2.3 Extraction of Common Targets

The disease-related targets overlapped with the active components of GFD. In this study, we obtained the intersecting genes related to GFD and KOA targets and generated a Venny map using the online Venny map platform [20]. The targets of active components of GFD related to KOA were then obtained for subsequent analysis.

### 2.4 Construction of the Compound-target Network

The compound-target network was visualized using Cytoscape (v.3.7.1) [21]. Information on the active components and their targets (common targets of diseases and active compounds of GFD described in Section 1.3) was imported into Cytoscape (v.3.7.1) to construct a compound-target interaction network to show the interaction relationships between the active components and targets. The software program was used to calculate network topology parameters, such as degree and betweenness centrality (BC), and thereby identify the main candidate targets and monomer components.

### 2.5 Construction of the Protein Interaction Network

Protein-protein interactions (PPIs) are the basis of cell function and play an important role in regulating the physiological and pathological state of the body. In PPI networks, nodes are often used to represent proteins, connection lines are used to represent PPIs, and the node size, colour, connection length, and thickness represent the topological parameters of the network [22]. To further understand the mechanism of the synergistic action between potential targets and disease targets of GFD at the protein level, a PPI relationship was obtained using Search Tool for the Retrieval of Interacting Genes/Proteins (STRING) (v.11.0) [23], and the core genes were then identified according to the number of node connections. In the analysis, the species was set to “*Homo sapiens*”, and high confidence = 0.9 was set as the lowest interaction score.

### 2.6 Gene Ontology (GO) and Kyoto Encyclopedia of Genes and Genomes (KEGG) Enrichment Analyses

The potential targets of GFD in the treatment of KOA (the common targets obtained as described in Section 1.3) were input into the list of target gene names through the Database for Annotation, Visualization, and Integrated Discovery (DAVID) (https://david.ncifcrf.gov/home.jsp) [24], the species was limited to humans, and the threshold value was set to *p* < 0.05. GO enrichment analysis and KEGG pathway annotation analysis of the potential targets of GFD in the treatment of cold-dampness obstruction-type KOA were conducted, and the important signalling pathway of GFD in the treatment of KOA was identified.

## 3 RESULTS

### 3.1 Active Constituents of GFD

A total of 620 types of chemical constituents of GFD, including 65, 220, 55, and 280 types of constituents of Fuzi, Guizhi, Baizhu and Gancao, respectively, were retrieved from the TCMSP. A total of 127 types of active ingredients of GFD, including 21, 7, 7, and 92 types of ingredients of Fuzi, Guizhi, Baizhu, and Gancao, respectively, were screened based on the criteria OB ≥ 30% and DL ≥ 0.18. Because 25 compounds had no corresponding targets, a subsequent analysis was not conducted. The analysis revealed 208 targets of the active ingredients (excluding repeated targets). Specific information on the selected active ingredients is shown in Table **1**.

### 3.2 Disease-related Targets of KOA

Using “osteoarthritis, knee” as the keywords, 4602 related disease targets were retrieved from the CTD, 2040 were retrieved from GeneCards and 368 were retrieved from DisGeNET. After merging the three datasets and removing duplicate items, a total of 5843 genes related to KOA were obtained.

### 3.3 Common Targets of Diseases and Compounds

**Fig. (1):**
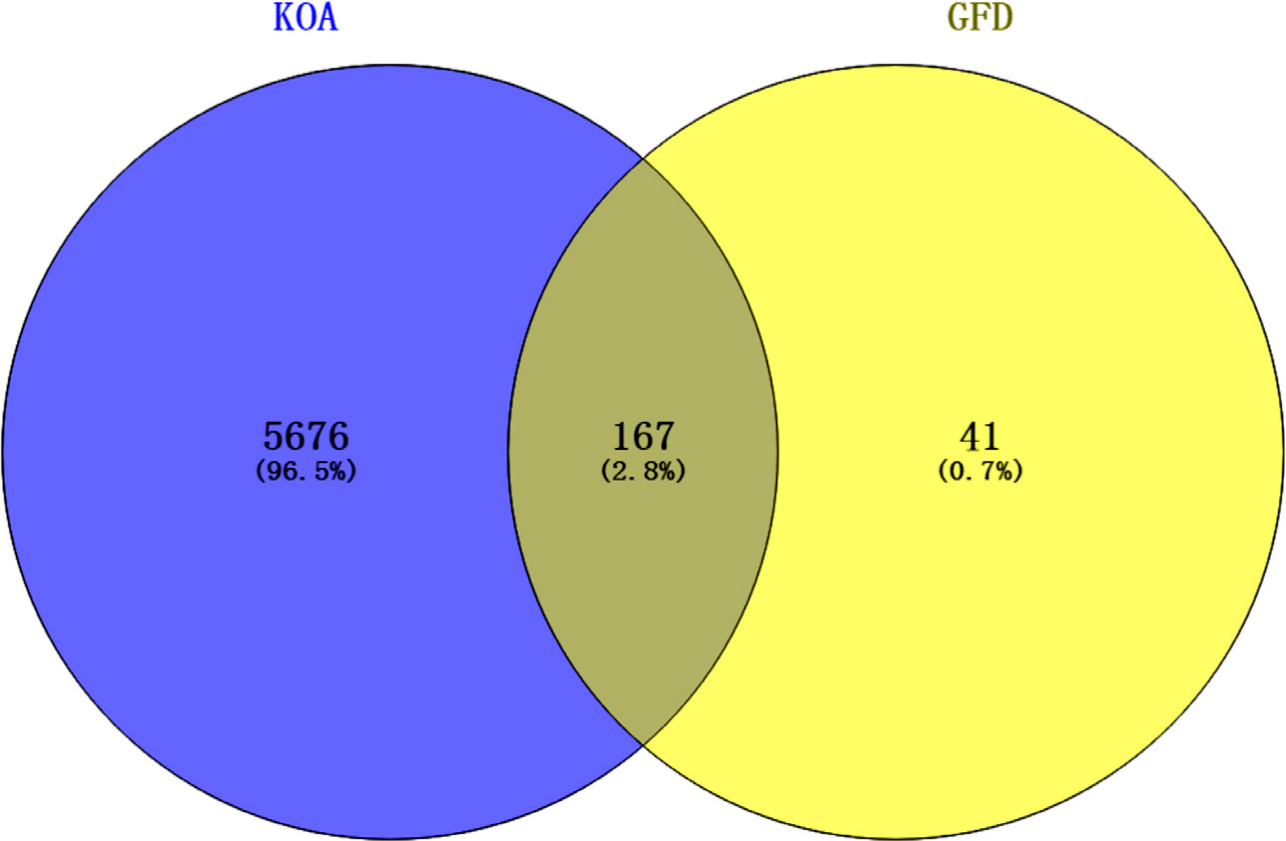
Venn diagram of KOA targets and GFD targets.

The active component target and disease target datasets were intersected, and a total of 167 targets of active components related to KOA were obtained for subsequent analysis (Fig. **1**). These 167 targets may be key in linking the therapeutic effect of GFD on KOA.

### 3.4 Construction and Analysis of the Compound-target Network

**Fig. (2):**
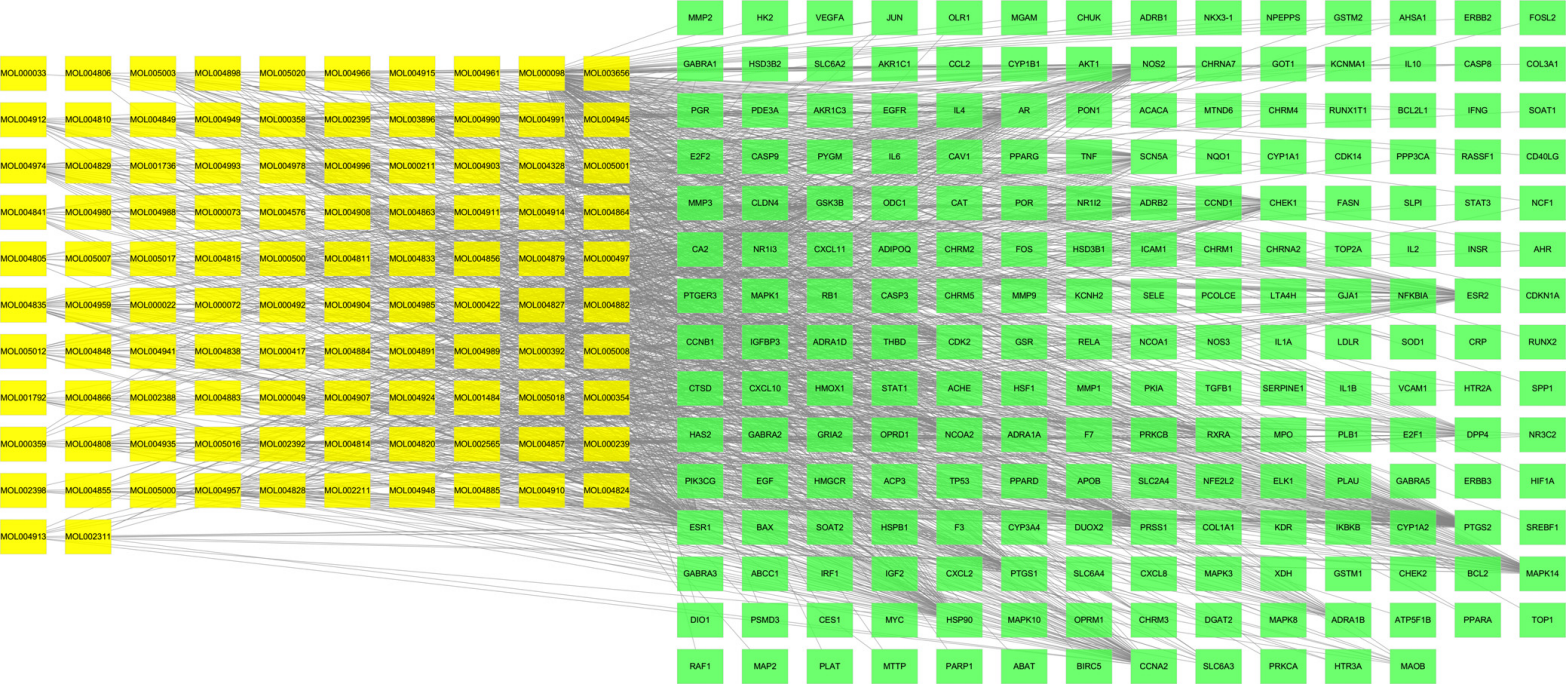
The gene target network of the effective components of GFD.

One hundred two active monomers and 208 targets of GFD were selected to construct the active component-target interaction network using Cytoscape (v.3.7.1). The yellow nodes represent active ingredients of GFD, and the green nodes are targets of the active ingredients. The network contained 2970 edges representing the interactions between targets and chemical components, which reflect the multi-component and multitarget characteristics of GFD (Fig. **2**). The network topology analysis showed that the network concentration, density, and heterogeneity were 0.402, 0.031, and 1.652, respectively. The average node degree was 9.58, and 96 nodes had above-average degrees. The average mediation centrality of the nodes was 0.006, and 42 nodes showed above-average mediation centrality. According to the topological properties of the degree value and intermediate centrality of the network nodes, the core nodes were selected and analysed. These nodes that connect the most compounds or targets play a key role in the whole network and may be key compounds or targets. Table **2** lists the 25 key nodes with above-average degree and centrality values and their topological parameters in the compound-target network.

### 3.5 Construction of the PPI Network

The 167 common targets obtained in Section 2.3 were inputted into STRING (v.11.0) for analysis, and the protein interaction results are shown in Fig. (**3**). The results from the PPI analysis revealed 148 nodes and 1430 edges. The nodes represent proteins, each edge represents a PPI relationship, and the average degree was 9.66. Fig. (**4**) shows a bar chart of the top 15 target targets in terms of degree value. These targets are considered the key targets of GFD in the treatment of cold-dampness obstruction-type KOA.

### 3.6 GO Enrichment Analysis

**Fig. (5):**
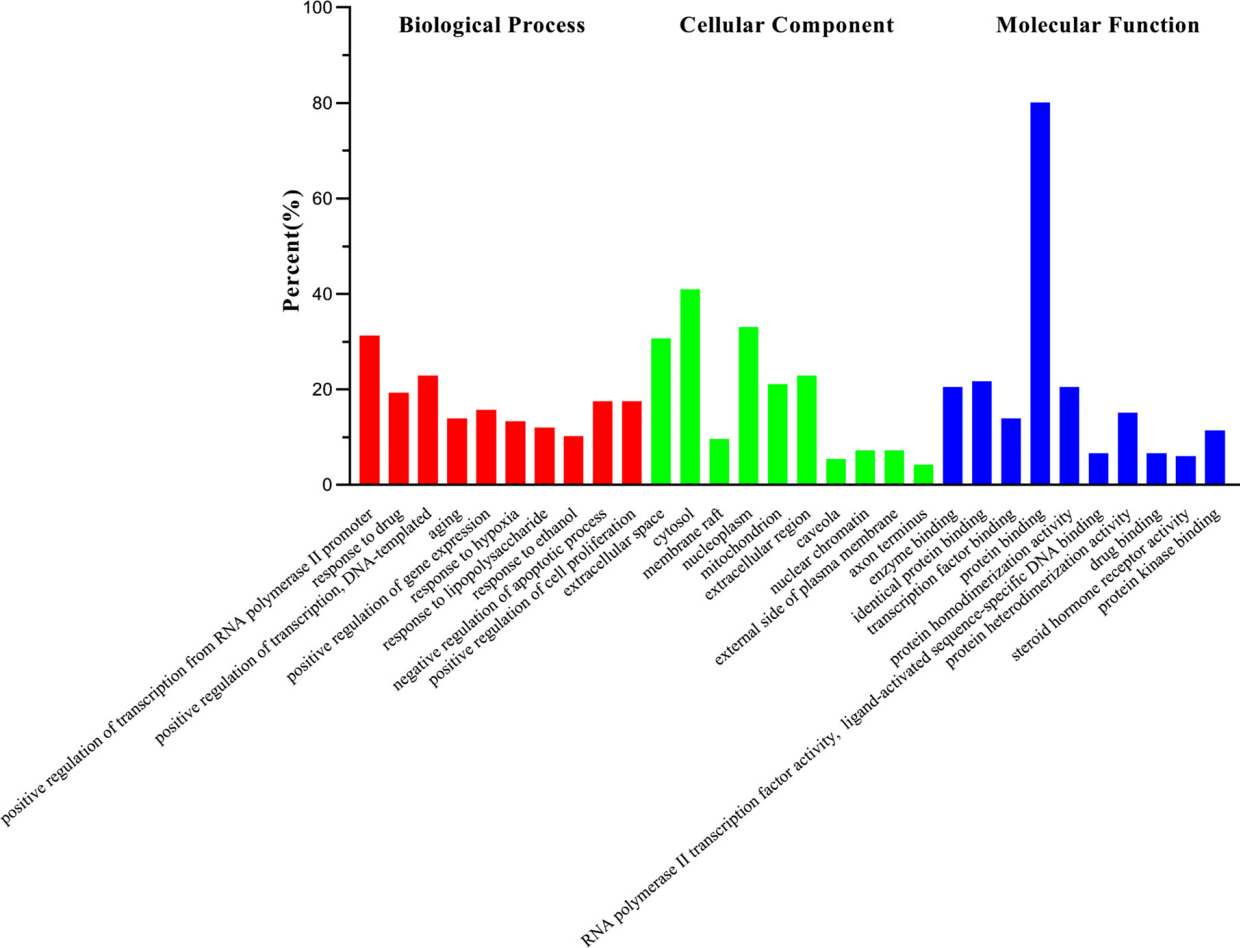
GO enrichment analysis of of GFD in the treatment KOA (top 10).

The 167 common targets of GFD and KOA were analysed by GO enrichment analysis, and 881 GO entries, including 685 biological processes (BP), 72 cellular component (CC) and 124 molecular functions (MF) terms, were obtained. According to the p-value and number of enriched genes, the top 10 BP, CC, and MF terms with high enrichment were selected to visualize and generate a bubble diagram (Fig. **5**). The main BP terms were related to positive regulation of transcription from the RNA polymerase II promoter, response to a drug, negative regulation of the apoptotic process, and inflammatory response, and the extracellular space, nucleoplasm, nuclear chromatin, and external side of plasma membrane were the main enriched CC terms. The main MF terms were related to identical protein binding, protein binding, and protein heterodimerization activity.

### 3.7 KEGG Pathway Enrichment Analysis

**Fig. (6):**
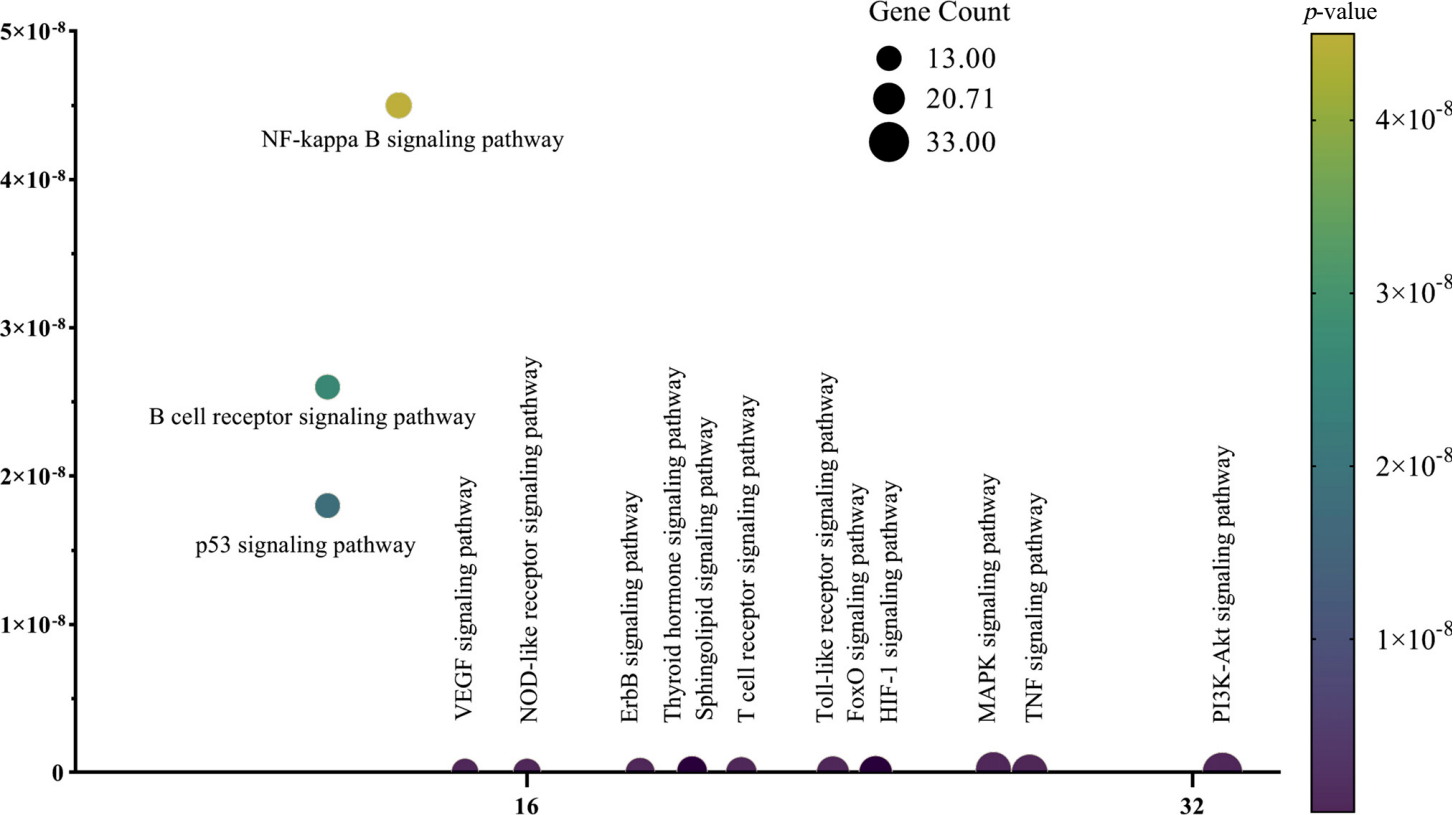
KEGG pathway enrichment analysis of of GFD in the treatment of KOA (top 15).

KEGG analysis of the intersecting targets of GFD and KOA showed that the key targets were enriched in 129 biological signalling pathways. According to the Benjamini‒Hochberg correction method and a *p*-value < 0.01, the top 15 noncancer disease pathways were analysed after p-value-based ranking, and a KEGG functional enrichment bubble chart was drawn (Fig. **6**). The main biological pathways identified in the analysis included the TNF signalling pathway, PI3K-Akt signalling pathway, VEGF signalling pathway, B-cell receptor signalling pathway, FoxO signalling pathway, and NF-kappa B signalling pathway.

## 4 DISCUSSION

KOA is a chronic degenerative osteoarthropathy with an increasing incidence rate. The incidence rate of KOA is approximately 4%-13% of the world’s population, and KOA has thus become a major disease affecting human health [25]. The quality of life of KOA patients is seriously affected by pain, dyskinesia, and other symptoms. Therefore, it is of great significance to explore more nonsurgical treatments to reduce the medical burden and improve the quality of life of these patients. TCM has unique advantages in the treatment of KOA. Cold-dampness obstruction syndrome is a common syndrome type of KOA, and GFD is a commonly used prescription for this syndrome type and has been proven to have good clinical effects [9, 26]. In this study, we used network pharmacology to explore the molecular mechanism of GFD in the treatment of cold-dampness obstruction syndrome-type KOA and found that it has multitarget, multi-component, and multichannel characteristics.

In this study, quercetin, kaempferol, beta-sitosterol, 7-methoxy-2-methyl isoflavone, and formononetin were found to be the core compounds of GFD. These core components regulate the TNF signalling pathway, MAPK signalling pathway, p53 signalling pathway, PI3K Akt signalling pathway, HIF-1 signalling pathway, T-cell receptor signalling pathway, and VEGF signalling pathway by affecting PTGS2, ESR1, HSP90, NOS2, JUN, MAPK1, MAPK3, TNF, TP53, IL6, EGFR, and other proteins to play a role in the treatment of cold-dampness obstruction-type KOA.

The pathogenesis of KOA is complex and involves many cytokines, proteins, and signalling pathways [27]. Articular cartilage is mainly composed of the extracellular matrix of the cartilage nucleus, and the cytokines regulating the function of KOA chondrocytes are mainly VEGF, TGF, IL, and TNF [28, 29]. An imbalance between the synthesis and metabolism of these inflammatory cytokines can cause the dysfunction of chondrocytes and eventually lead to the destruction of the cartilage matrix and loss of joint function [28-30]. The TNF signalling pathway plays a key role in immune regulation, the inflammatory response, cell differentiation, and apoptosis and is a classic inflammatory pathway [31]. This study found that the key targets of the active ingredients of GFD are PTGS2, TNF, IL6, and other inflammation-related proteins, which may indicate that GFD can intervene in cold-dampness obstruction-type KOA by regulating inflammatory factors to alter TNF signalling.

In osteoarticular diseases, p53 can block the cell cycle, promote apoptosis and accelerate cartilage degradation by inhibiting DNA replication [32, 33]. Studies have shown that the expression of p53 in osteoarthritis chondrocytes is higher than that in normal chondrocytes. Therefore, downregulating the expression of p53 can reduce chondrocyte apoptosis and prevent and alleviate osteoarthritis [34]. GFD contains flavonoids, quinones, and sterols, which can play an anti-inflammatory role by inhibiting the MAPK signalling pathway [35]. The PI3K-Akt signalling pathway is a classic antiapoptotic pathway [36] that plays an important role in cell differentiation, proliferation, apoptosis and glucose transport. Moreover, this pathway can achieve rapid signal transduction from the membrane to the nucleus, regulate chondrocyte proliferation, apoptosis, and matrix remodelling, and play a prominent role in the process of chondrocyte apoptosis [37]. The PI3K/Akt signalling pathway can be activated in various ways at the early stage of osteoarthritis, and its downstream pathways involving caspases and NF-κB can participate in a variety of processes, including cell proliferation, apoptosis, and differentiation regulation; in addition, the PI3K/Akt pathway is an important pathway for inhibiting chondrocyte apoptosis in KOA [38]. This study also preliminarily verified that GFD could delay the progression of KOA by regulating PI3K-Akt signalling pathway-related proteins.

Although this study presents several findings, there remain some defects. On the one hand, the lack of TCM syndrome-related gene target databases affects the accuracy of network pharmacology in predicting the mechanism of action, target, and pathway of Chinese herbal medicines. On the other hand, the current drug data focus on the interactions between drugs for the prevention and treatment of diseases, but research methods based on network pharmacology cannot reveal the synergistic effects of Chinese herbal medicine, which is a field that needs to be further explored in the future.

## CONCLUSION

Based on network pharmacology, this study linked the core components of GFD with KOA-related targets of cold-dampness obstruction syndrome and intuitively showed the biological pathway and targets of GFD in the treatment of KOA. This study revealed that GFD may play a role in the treatment of cold-dampness obstruction syndrome-type KOA by inhibiting the release of related inflammatory factors and suppressing chondrocyte apoptosis through related pathways and targets. By applying network pharmacology, we screened the commonly used compounds for the treatment of this disease and preliminarily identified the correlation between the active components of GFD and cold-dampness obstruction syndrome-type KOA, which can provide certain directions and ideas for future experimental research on GFD.

## Figures and Tables

**Fig. (3):**
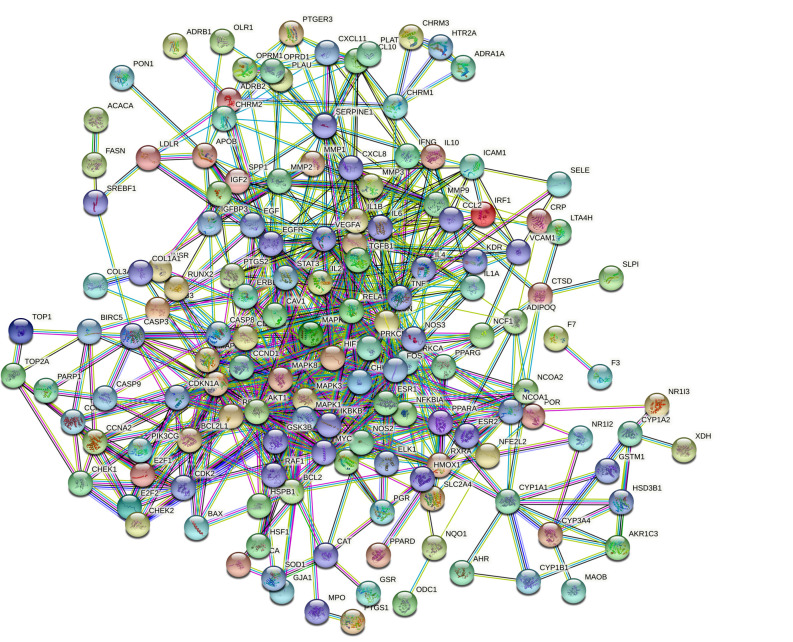
PPI network diagram of target protein.

**Fig. (4):**
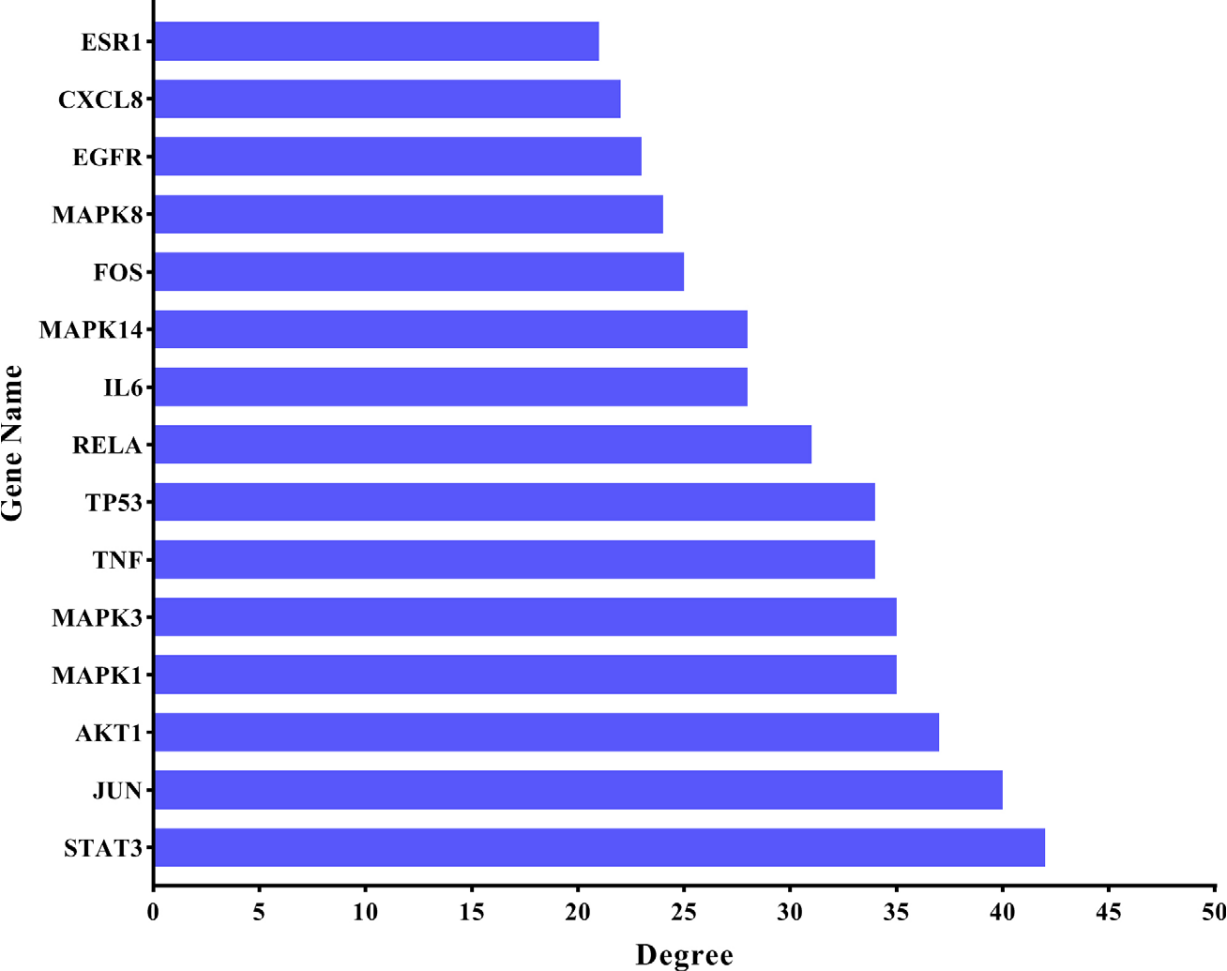
Core targets of GFD in the treatment of KOA (top 15).

**Table 1:** Information on active ingredients of GFD.

**Herb Name**	**Molecule ID**	**Molecule Name**	**OB (%)**	**DL**
Fu Zi	MOL002211	11,14-eicosadienoic acid	39.99	0.2
Fu Zi	MOL002388	Delphin_qt	57.76	0.28
Fu Zi	MOL002392	Deltoin	46.69	0.37
Fu Zi	MOL002395	Deoxyandrographolide	56.3	0.31
Fu Zi	MOL002398	Karanjin	69.56	0.34
Fu Zi	MOL000359	sitosterol	36.91	0.75
Bai Zhu	MOL000022	14-acetyl-12-senecioyl-2E,8Z,10E-atractylentriol	63.37	0.3
Bai Zhu	MOL000033	(3S,8S,9S,10R,13R,14S,17R)-10,13-dimethyl-17-[(2R,5S)-5-propan-2-yloctan-2-yl]-2,3,4,7,8,9,11,12,14,15,16,17-dodecahydro-1H-cyclopenta[a]phenanthren-3-ol	36.23	0.78
Bai Zhu	MOL000049	3β-acetoxyatractylone	54.07	0.22
Bai Zhu	MOL000072	8β-ethoxy atractylenolide III	35.95	0.21
Gui Zhi	MOL001736	(-)-taxifolin	60.51	0.27
Gui Zhi	MOL000358	beta-sitosterol	36.91	0.75
Gui Zhi	MOL000359	sitosterol	36.91	0.75
Gui Zhi	MOL000492	(+)-catechin	54.83	0.24
Gui Zhi	MOL000073	ent-Epicatechin	48.96	0.24
Gui Zhi	MOL004576	taxifolin	57.84	0.27
Gan Cao	MOL001484	Inermine	75.18	0.54
Gan Cao	MOL001792	DFV	32.76	0.18
Gan Cao	MOL000211	Mairin	55.38	0.78
Gan Cao	MOL002311	Glycyrol	90.78	0.67
Gan Cao	MOL000239	Jaranol	50.83	0.29
Gan Cao	MOL002565	Medicarpin	49.22	0.34
Gan Cao	MOL000354	isorhamnetin	49.6	0.31
Gan Cao	MOL000359	sitosterol	36.91	0.75
Gan Cao	MOL003656	Lupiwighteone	51.64	0.37
Gan Cao	MOL003896	7-Methoxy-2-methyl isoflavone	42.56	0.2
Gan Cao	MOL000392	formononetin	69.67	0.21
Gan Cao	MOL000417	Calycosin	47.75	0.24
Gan Cao	MOL000422	kaempferol	41.88	0.24
Gan Cao	MOL004328	naringenin	59.29	0.21
Gan Cao	MOL004805	(2S)-2-[4-hydroxy-3-(3-methylbut-2-enyl)phenyl]-8,8-dimethyl-2,3-dihydropyrano [2,3-f]chromen-4-one	31.79	0.72
Gan Cao	MOL004806	euchrenone	30.29	0.57
Gan Cao	MOL004808	glyasperin B	65.22	0.44
Gan Cao	MOL004810	glyasperin F	75.84	0.54
Gan Cao	MOL004811	Glyasperin C	45.56	0.4
Gan Cao	MOL004814	Isotrifoliol	31.94	0.42
Gan Cao	MOL004815	(E)-1-(2,4-dihydroxyphenyl)-3-(2,2-dimethylchromen-6-yl)prop-2-en-1-one	39.62	0.35
Gan Cao	MOL004820	kanzonols W	50.48	0.52
Gan Cao	MOL004824	(2S)-6-(2,4-dihydroxyphenyl)-2-(2-hydroxypropan-2-yl)-4-methoxy-2,3-dihydrofuro [3,2-g]chromen-7-one	60.25	0.63
Gan Cao	MOL004827	Semilicoisoflavone B	48.78	0.55
Gan Cao	MOL004828	Glepidotin A	44.72	0.35
Gan Cao	MOL004829	Glepidotin B	64.46	0.34
Gan Cao	MOL004833	Phaseolinisoflavan	32.01	0.45
Gan Cao	MOL004835	Glypallichalcone	61.6	0.19
Gan Cao	MOL004838	8-(6-hydroxy-2-benzofuranyl)-2,2-dimethyl-5-chromenol	58.44	0.38
Gan Cao	MOL004841	Licochalcone B	76.76	0.19
Gan Cao	MOL004848	licochalcone G	49.25	0.32
Gan Cao	MOL004849	3-(2,4-dihydroxyphenyl)-8-(1,1-dimethylprop-2-enyl)-7-hydroxy-5-methoxy-coumarin	59.62	0.43
Gan Cao	MOL004855	Licoricone	63.58	0.47
Gan Cao	MOL004856	Gancaonin A	51.08	0.4
Gan Cao	MOL004857	Gancaonin B	48.79	0.45
Gan Cao	MOL004863	3-(3,4-dihydroxyphenyl)-5,7-dihydroxy-8-(3-methylbut-2-enyl)chromone	66.37	0.41
Gan Cao	MOL004864	5,7-dihydroxy-3-(4-methoxyphenyl)-8-(3-methylbut-2-enyl)chromone	30.49	0.41
Gan Cao	MOL004866	2-(3,4-dihydroxyphenyl)-5,7-dihydroxy-6-(3-methylbut-2-enyl)chromone	44.15	0.41
Gan Cao	MOL004879	Glycyrin	52.61	0.47
Gan Cao	MOL004882	Licocoumarone	33.21	0.36
Gan Cao	MOL004883	Licoisoflavone	41.61	0.42
Gan Cao	MOL004884	Licoisoflavone B	38.93	0.55
Gan Cao	MOL004885	licoisoflavanone	52.47	0.54
Gan Cao	MOL004891	shinpterocarpin	80.3	0.73
Gan Cao	MOL004898	(E)-3-[3,4-dihydroxy-5-(3-methylbut-2-enyl)phenyl]-1-(2,4-dihydroxyphenyl)prop-2-en-1-one	46.27	0.31
Gan Cao	MOL004903	liquiritin	65.69	0.74
Gan Cao	MOL004904	licopyranocoumarin	80.36	0.65
Gan Cao	MOL004907	Glyzaglabrin	61.07	0.35
Gan Cao	MOL004908	Glabridin	53.25	0.47
Gan Cao	MOL004910	Glabranin	52.9	0.31
Gan Cao	MOL004911	Glabrene	46.27	0.44
Gan Cao	MOL004912	Glabrone	52.51	0.5
Gan Cao	MOL004913	1,3-dihydroxy-9-methoxy-6-benzofurano [3,2-c]chromenone	48.14	0.43
Gan Cao	MOL004914	1,3-dihydroxy-8,9-dimethoxy-6-benzofurano [3,2-c]chromenone	62.9	0.53
Gan Cao	MOL004915	Eurycarpin A	43.28	0.37
Gan Cao	MOL004924	(-)-Medicocarpin	40.99	0.95
Gan Cao	MOL004935	Sigmoidin-B	34.88	0.41
Gan Cao	MOL004941	(2R)-7-hydroxy-2-(4-hydroxyphenyl)chroman-4-one	71.12	0.18
Gan Cao	MOL004945	(2S)-7-hydroxy-2-(4-hydroxyphenyl)-8-(3-methylbut-2-enyl)chroman-4-one	36.57	0.32
Gan Cao	MOL004948	Isoglycyrol	44.7	0.84
Gan Cao	MOL004949	Isolicoflavonol	45.17	0.42
Gan Cao	MOL004957	HMO	38.37	0.21
Gan Cao	MOL004959	1-Methoxyphaseollidin	69.98	0.64
Gan Cao	MOL004961	Quercetin der.	46.45	0.33
Gan Cao	MOL004966	3'-Hydroxy-4'-O-Methylglabridin	43.71	0.57
Gan Cao	MOL000497	licochalcone a	40.79	0.29
Gan Cao	MOL004974	3'-Methoxyglabridin	46.16	0.57
Gan Cao	MOL004978	2-[(3R)-8,8-dimethyl-3,4-dihydro-2H-pyrano [6,5-f]chromen-3-yl]-5-methoxyphenol	36.21	0.52
Gan Cao	MOL004980	Inflacoumarin A	39.71	0.33
Gan Cao	MOL004985	icos-5-enoic acid	30.7	0.2
Gan Cao	MOL004988	Kanzonol F	32.47	0.89
Gan Cao	MOL004989	6-prenylated eriodictyol	39.22	0.41
Gan Cao	MOL004990	7,2',4'-trihydroxy-5-methoxy-3-arylcoumarin	83.71	0.27
Gan Cao	MOL004991	7-Acetoxy-2-methylisoflavone	38.92	0.26
Gan Cao	MOL004993	8-prenylated eriodictyol	53.79	0.4
Gan Cao	MOL004996	gadelaidic acid	30.7	0.2
Gan Cao	MOL000500	Vestitol	74.66	0.21
Gan Cao	MOL005000	Gancaonin G	60.44	0.39
Gan Cao	MOL005001	Gancaonin H	50.1	0.78
Gan Cao	MOL005003	Licoagrocarpin	58.81	0.58
Gan Cao	MOL005007	Glyasperins M	72.67	0.59
Gan Cao	MOL005008	Glycyrrhiza flavonol A	41.28	0.6
Gan Cao	MOL005012	Licoagroisoflavone	57.28	0.49
Gan Cao	MOL005016	Odoratin	49.95	0.3
Gan Cao	MOL005017	Phaseol	78.77	0.58
Gan Cao	MOL005018	Xambioona	54.85	0.87
Gan Cao	MOL005020	dehydroglyasperins C	53.82	0.37
Gan Cao	MOL000098	quercetin	46.43	0.28

**Table 2:** Key node of compound-target network and its topological feature.

**Node**	**Node Type**	**Degree**	**BC**	**Node**	**Node Type**	**Degree**	**BC**
MOL000098	Monomer	133	0.52201214	SCN5A	Target	46	0.02712635
PTGS2	Target	93	0.13216659	DPP4	Target	36	0.02102651
ESR1	Target	80	0.03767813	MOL000358	Monomer	35	0.05926428
HSP90	Target	68	0.07961992	MOL003896	Monomer	35	0.02596826
AR	Target	67	0.04733605	ADRB2	Target	31	0.01880802
NOS2	Target	65	0.01885268	MOL000392	Monomer	31	0.04104519
GSK3B	Target	56	0.00816985	MOL002565	Monomer	30	0.02470553
PRSS1	Target	55	0.03335627	MOL004328	Monomer	30	0.10979428
ESR2	Target	54	0.00868787	RXRA	Target	30	0.01684741
CCNA2	Target	53	0.00795874	MOL000354	Monomer	27	0.02757089
MOL000422	Monomer	53	0.10904982	MOL000497	Monomer	26	0.02968868
NCOA2	Target	51	0.06353307	MOL000500	Monomer	26	0.00952649
PTGS1	Target	48	0.05247322	-	-	-	-

## Data Availability

The datasets analyzed in this study were obtained from the corresponding author (LZ and JL) upon reasonable request or open-access online databases.

## LIST OF ABBREVIATIONS

CTD: Comparative Toxicogenomics Database
DAVID: Database for Annotation, Visualization and Integrated Discovery
GFD: *Gancao fuzi* Decoction
KEGG: Kyoto Encyclopedia of Genes and Genomes.
KOA: knee Osteoarthritis
TCMSP: Traditional Chinese Medicine Systems Pharmacology
